# Adiposity and breast cancer risk in postmenopausal women: Results from the UK Biobank prospective cohort

**DOI:** 10.1002/ijc.31394

**Published:** 2018-04-10

**Authors:** Wenji Guo, Timothy J. Key, Gillian K. Reeves

**Affiliations:** ^1^ Cancer Epidemiology Unit, Nuffield Department of Population Health University of Oxford Oxford United Kingdom

**Keywords:** breast cancer, adiposity, obesity, bioelectrical impedance, UK Biobank

## Abstract

Body size is an important modifiable risk factor for postmenopausal breast cancer. However, it remains unclear whether direct measures of fat mass are better indicators of risk than anthropometric measures, or whether central adiposity may contribute to risk beyond overall adiposity. We analyzed data from 162,691 postmenopausal women in UK Biobank followed from 2006 to 2014. Body size was measured by trained technicians. Multivariable‐adjusted Cox regression was used to estimate relative risks. Analyses were stratified by age at recruitment, region and socioeconomic status, and adjusted for family history of breast cancer, age at menarche, age at first birth, parity, age at menopause, previous hormone replacement therapy use, smoking, alcohol intake, height, physical activity and ethnicity. We observed 2,913 incident invasive breast cancers during a mean 5.7 years of follow‐up. There was a continuous increase in risk of postmenopausal breast cancer with increasing adiposity, across all measures. The point estimate, comparing women in the top (median 37.6 kg) to bottom (median 17.6 kg) quartile of body fat mass was 1.70 (95% confidence interval 1.52–1.90). The magnitudes of the associations between per SD increase in BMI and body fat mass with breast cancer risk were similar, suggesting impedance measures of fat were not substantially better indicators of risk than anthropometric measures. After adjusting for body fat mass, the associations between anthropometric measures of central adiposity and breast cancer risk were attenuated. The magnitude of risk, across all measures of adiposity, was greater in women who had been postmenopausal for 12 or more years.

## Abstract 2

**What's new?**

Obesity is associated with an increased risk of postmenopausal breast cancer. However, whether direct anaylses of fat mass using bioelectrical impedance measures are better indicators of breast cancer risk than anthropometric measures, such as body mass index (BMI) and waist circumference, remains unclear. In this study, based on data for postmenopausal women obtained from UK Biobank, the authors found that BMI, waist circumference, and direct bioimpedance measures of fat showed similarly strong associations with postmenopausal breast cancer risk. Central adiposity was no longer associated with breast cancer risk after adjusting for overall body fat mass.

**Abbreviations**
BMI: body mass index
CI: confidence interval
DXA: dual‐energy X‐ray absorptiometry
HRT: hormone replacement therapy
RR: relative risk

## Introduction

Although there are several important known risk factors for breast cancer, excess adiposity is one of the few modifiable ones. Previous studies have demonstrated an association between obesity and increased risk for postmenopausal breast cancer,^1^ but questions remain about this relationship and its underlying mechanisms. Since adipose tissue is the major site of estrogen synthesis in postmenopausal women, the most prominent hypothesis is that more body fat is linked to increased postmenopausal breast cancer risk through estrogen‐stimulated carcinogenesis.^2^, ^3^ For this reason, fat mass may be a better predictor of postmenopausal breast cancer risk than other measures of adiposity.

Due to high cost and difficulty of implementation, most large prospective cohort studies have been unable to directly address this question, instead examining the association between obesity and breast cancer risk using only traditional anthropometric measures such as body mass index (BMI), waist circumference and waist‐to‐hip ratio, which do not directly distinguish between lean and fat mass.^4^, ^5^, ^6^, ^7^ While BMI is unable to capture changes in adiposity with age, bioelectrical impedance measures reflect increases in fat mass with age.^8^ Furthermore, it is unclear whether the distribution of adipose tissue is relevant for breast cancer risk.^9^ Most studies rely on self‐reported measures of body size, which tend to underestimate adiposity.^10^ We aim here to clarify the association between obesity and postmenopausal breast cancer risk using data on bioelectrical impedance measures of body fat in UK Biobank, a nationwide study of 500,000 individuals.

## Methods

### Data source

Data were obtained from UK Biobank (reference number 3248, approved August 2013). Details of the rationale, design and survey methods for UK Biobank have been described elsewhere^11^ and information on data available and access procedures are given on the study website (http://www.ukbiobank.ac.uk/). UK Biobank has approval from the North West Multi‐centre Research Ethics Committee, the Confidentiality Advisory Group in England and Wales and the Community Health Index Advisory Group in Scotland. All participants provided written informed consent.

### Study participants

The complete UK Biobank dataset includes 502,620 UK adults (229,165 men and 273,455 women) aged 40–70 at recruitment during 2006–2010. Participants completed a touchscreen questionnaire during the baseline assessment center visit that included questions on socio‐demographics, lifestyle, health and medical history and sex‐specific factors. A number of physical measurements, including body size and composition, were also assessed on the whole cohort during the baseline assessment center visit. A repeat assessment of all baseline measures was conducted in 20,345 participants at the UK Biobank Coordinating Centre between August 2012 and June 2013.

Women were eligible for these analyses if they were postmenopausal at recruitment. Women were defined as being postmenopausal at recruitment if they reported that their periods had stopped. Women with unknown self‐reported menopausal status were defined as postmenopausal if they were aged 53 or over at recruitment based on previously established criteria^12^ and because >97% of the study population with known menopausal status reported having become postmenopausal by that age. Women were excluded if they had a prior cancer diagnosis (except for non‐melanoma skin cancer ICD‐10 C44) (*n* = 18,372), were premenopausal (*n* = 62,899) or had unknown menopausal status after applying the above categorization criteria (*n* = 12,563), were current users of hormone replacement therapy (HRT) (*n* = 13,680), or had missing data on any measure of body size and composition (*n* = 3,250). Current HRT users were excluded because HRT use is known to attenuate associations of adiposity with breast cancer risk in postmenopausal women.^4^, ^13^, ^14^ 162,691 postmenopausal women were included in the analyses (Supporting Information Fig. S1).

### Anthropometry and body composition

At the UK Biobank baseline interview, trained staff measured standing height using the Seca 202 device (Seca, Hamburg, Germany). The Wessex non‐stretchable sprung tape measure (Wessex, United Kingdom) was used to measure waist circumference and hip circumference, from which we derived waist‐to‐hip‐ratio by dividing waist circumference by hip circumference. The Tanita BC‐418MA body composition analyzer (Tanita, Tokyo, Japan) was used to measure body and trunk fat mass and percentage using bio‐impedance. BMI was calculated by dividing weight (kg) by the square of standing height (m^2^). Dual‐energy X‐ray absorptiometry (DXA) was used to measure fat mass/percentage on a subset of participants beginning in 2014 using the GE‐Lunar iDXA (GE Healthcare, Chicago, IL).

### Ascertainment of cancer cases

UK Biobank obtains data on cancer diagnoses through the Health & Social Care Information Centre for participants in England and Wales, and the NHS Central Register for participants in Scotland.

### Statistical analyses

Baseline characteristics of study participants were compared between top and bottom quartiles of BMI, body fat mass and waist circumference. Associations between the various measures of body size and composition were examined by calculating age‐adjusted Pearson's partial correlation coefficients.

Women were followed from date of baseline assessment center visit until the earliest of: date of breast cancer registration (ICD‐10 C50), date of death, date of loss to follow‐up or end of follow‐up for cancer incidence (November 30, 2014). Women diagnosed with any cancer other than breast cancer (with the exception of non‐melanoma skin cancer) during follow‐up were censored at date of diagnosis.

Multivariable‐adjusted Cox regression with attained age as the underlying time variable was used to estimate hazards ratios (referred to as relative risks [RRs]) and 95% confidence intervals (CIs) for the association between breast cancer risk and body size and composition measures: height, weight, BMI, body fat mass, body fat percentage, waist circumference, hip circumference, waist‐to‐hip ratio, trunk fat mass and trunk fat percentage. For the risk analyses, women were categorized into quartiles according to their baseline body size/composition. To facilitate comparison across measures of adiposity, we also estimated RRs per standard deviation (SD) increase and show the results from tests for linear trend corrected using the repeat assessment median within each category (grouped into quartiles) and the corresponding *χ* ^2^ statistic. Tests for linear trend were performed with categories coded in an ordinal fashion using the repeat assessment median of each category to attain measures that are more representative of the true long‐term values.^15^ In a separate analysis, tests for linear trend were performed using the DXA median of each quartile for fat mass/percentage variables.

All analyses were stratified by 5‐year age at recruitment categories, region of recruitment and socioeconomic status (based on quintiles of Townsend deprivation index),^16^ which allows the hazard function to vary across levels of the stratification variables. All analyses were adjusted for family history of breast cancer (no, yes), age at menarche (<12, 12–13, ≥14), age at first birth (<25, 25–29, ≥30), parity (nulliparous, 1–2, ≥3), age at menopause (<45, 45–54, ≥55), previous HRT use (never, past), smoking (never, past, current), alcohol intake frequency (<3 times a month, 1–4 times a week, daily or almost daily), physical activity (<14, 14–30, 31–59.8, ≥59.9 metabolic equivalent hours per week) and ethnicity (White, Mixed, Asian or Asian British, Black or Black British, Chinese, other ethnic group). Regression models with exposures other than height as the exposure of interest were adjusted for height (<160, 160–165.9, ≥166 cm) as an independent risk factor for breast cancer. Analyses were further adjusted for body size at age 10 (thinner, about average, plumper) to account for the independent association between greater childhood body fatness and decreased breast cancer risk.^17^ Women with missing values for any of the adjustment variables were assigned to a separate “unknown” category for the respective variable. Information was either missing or reported as unknown for <3% of covariates, with the exception of total physical activity (28.5%) and age at menopause (18.7%) which may be difficult to accurately report if menopausal status is masked by hysterectomy or HRT before menstrual periods stop naturally.^12^ To assess the impact of missing values, we conducted a sensitivity analysis restricted to participants with known values for all adjustment variables.

The *χ* ^2^ statistic for trend across quartile medians was used to quantify the extent to which each particular index of adiposity was related to risk.^18^ We further adjusted anthropometric measures of central adiposity by the impedance measure of body fat mass, but we chose not to mutually adjust impedance measures for each other, because these variables were highly correlated (*r* >0.88). Assessment of interaction terms between each exposure of interest and the underlying time variable did not suggest any significant deviation from proportional hazards.

RRs were also estimated for each body size/composition variable as a function of years since menopause (<12, ≥12) by using a time‐varying covariate. Comparisons were made between women who were <12 years and ≥12 years since menopause because this cut point divided the number of cases approximately equally. Likelihood ratio tests were used to assess whether time since menopause was an effect modifier in the associations between body size/composition variables and breast cancer risk. All analyses were conducted using STATA version 15.0 (Stata Corp LP, College Station, TX).

## Results

Two thousand nine hundred and thirteen invasive breast cancer cases were diagnosed among 162,691 postmenopausal women at risk during a mean follow‐up of 5.7 (SD 1.1) years. Table 1 shows baseline characteristics of participants according to BMI, body fat mass and waist circumference. Women in the top quartiles for these body size/composition variables were more likely to be in the lowest fifth of socioeconomic class, have a younger age at first birth, have used HRT, consume alcohol only occasionally or never and engage in less physical activity while the leanest women were more likely to have used oral contraceptives in the past. Those in the bottom quartiles of BMI and body fat mass were more likely to be either never or current smokers.

**Table 1:** Baseline characteristics for the 162,691 postmenopausal participants of UK Biobank by various measures of adiposity

	Body mass index (kg/m^2^)		Body fat mass (kg)		Waist circumference (cm)	
	Quartile 1	Quartile 4	Quartile 1	Quartile 4	Quartile 1	Quartile 4
Age at recruitment, mean (SD)	59.8 (5.4)	60.3 (5.4)	59.9 (5.4)	60.3 (5.4)	59.6 (5.4)	60.6 (5.3)
Lowest socioeconomic quintile, %	16	25.1	16.7	23.8	15.2	25.5
Family history of breast cancer, %	7.2	6.1	6.8	6.3	7	6.2
Age at menarche, mean (SD)	13.2 (1.6)	12.7 (1.7)	13.1 (1.6)	12.7 (1.7)	13.1 (1.6)	12.7 (1.7)
Age at first birth, mean (SD)	26.4 (4.9)	24.6 (4.8)	26.2 (4.9)	24.7 (4.9)	26.2 (4.9)	24.7 (4.9)
Parity, mean (SD)	1.8 (1.2)	2.0 (1.3)	1.8 (1.2)	2.0 (1.2)	1.8 (1.1)	2.0 (1.3)
Ever oral contraceptive use, %	78.8	75.3	78.3	76.2	79	75.1
Age at menopause, mean (SD)	49.9 (4.6)	49.6 (5.5)	49.9 (4.7)	49.6 (5.4)	49.9 (4.6)	49.6 (5.4)
Past hormone replacement therapy use, %	43.1	47.6	42.6	47.8	42.7	47.5
*Smoking, %*						
Never	61	56.5	61.1	55.7	62.6	54.5
Past	29.7	35.6	29.3	36.5	29.1	36.6
Current	9.1	7.4	9.4	7.2	8	8.4
*Alcohol intake frequency, %*						
Special occasions only or never	21.1	36.2	22.2	34.4	21.3	35.8
1–3 times a month	10.3	15.1	10.6	14.8	11	14.4
1–4 times a week	46.3	38.4	45.8	39.6	47.5	38
Daily or almost daily	22.2	10.3	21.3	11.2	20.1	11.7
Height (cm), mean (SD)	162.9 (6.2)	160.8 (6.1)	160.5 (6.2)	162.9 (6.1)	161.5 (6.1)	162.0 (6.2)
Total physical activity (MET‐hr), mean (SD)	53.3 (56.8)	40.3 (50.2)	55.1 (58.7)	39.0 (48.4)	53.9 (57.0)	39.6 (49.3)

Table 2 shows age‐adjusted Pearson's partial correlation coefficients for the various anthropometric and body composition measures. BMI was highly correlated with both body fat percentage (0.85) and body fat mass (0.94). Among the indicators of central obesity, trunk fat percentage and mass were highly correlated with waist circumference (0.74 and 0.83 respectively) and hip circumference (0.75 and 0.86 respectively) but not with waist‐to‐hip ratio (0.39 and 0.41 respectively). Waist‐to‐hip ratio was much more strongly correlated with waist circumference (0.74) than hip circumference (0.23). Total and central adiposity were very highly correlated as shown by the correlation coefficients between body and trunk fat percentage (0.98) and body and trunk fat mass (0.97). In view of these high correlations between certain indices, associations of risk with overall fatness were not mutually adjusted for other indices of body fatness. We present results for both body and trunk fat mass, but the extremely high correlation coefficients observed between these two indices indicate that their effects may be indistinguishable.

**Table 2:** Age‐adjusted Pearson's partial correlation coefficients for the 162,691 postmenopausal participants

	Height, cm	Weight, kg	BMI, kg/m^2^	Waist circumference, cm	Hip circumference, cm	Waist‐to‐hip ratio	Body fat mass, kg	Body fat %	Trunk fat mass, kg	Trunk fat %
Height, cm	1.00									
Weight, kg	0.27	1.00								
BMI, kg/m^2^	−0.13	0.92	1.00							
Waist circumference, cm	0.04	0.86	0.87	1.00						
Hip circumference, cm	0.12	0.91	0.89	0.82	1.00					
Waist‐to‐hip ratio	−0.07	0.41	0.45	0.74	0.23	1.00				
Body fat mass, kg	0.15	0.97	0.94	0.88	0.91	0.43	1.00			
Body fat %	0.01	0.83	0.85	0.81	0.81	0.44	0.93	1.00		
Trunk fat mass, kg	0.24	0.92	0.85	0.83	0.86	0.41	0.97	0.94	1.00	
Trunk fat %	0.13	0.77	0.75	0.74	0.75	0.39	0.88	0.98	0.94	1.00

**Figure 1:**
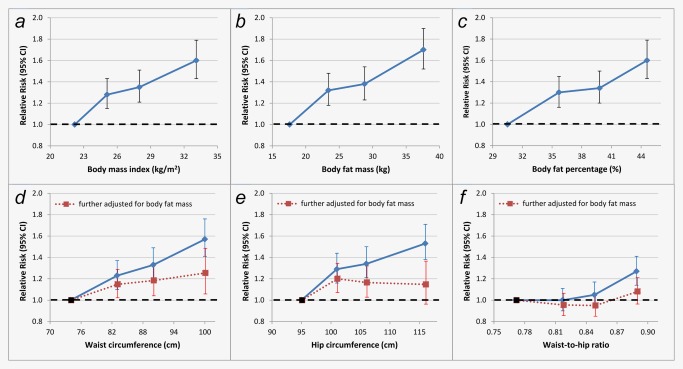
Association between various measures of adiposity and postmenopausal breast cancer risk: (*a*) body mass index, (*b*) body fat mass, (*c*) body fat percentage, (*d*) waist circumference, (*e*) hip circumference, (*f*) waist‐to‐hip ratio. Analyses are adjusted for family history of breast cancer, age at menarche, age at first birth, parity, age at menopause, previous HRT use, smoking, alcohol intake frequency, physical activity, ethnicity, height and body size at age 10. For panels *d*, *e* and *f*, the blue diamonds connected by a solid lines represent multivariable adjusted estimates and the red squares connected by dotted lines represent estimates further adjusted for body fat mass. Body size/composition measures were grouped into quartiles. The figure shows point estimates and 95% confidence intervals. [Color figure can be viewed at http://wileyonlinelibrary.com]

All measures of overall adiposity, as well as height, were associated with increased postmenopausal breast cancer risk in an approximately linear fashion (Fig. 1, Table 3). After adjusting for body size at age 10, the magnitudes of association for all indices of adiposity were increased. Lowest breast cancer risk was associated with being in the lowest quartile of adiposity (median BMI 22.2 kg/m^2^). Women in the top quartile of body fat mass (32.6–108.4 kg; median, 37.6 kg), had the greatest increase in breast cancer risk (RR, 1.70; 95% CI, 1.52–1.90; *p*_trend_ <0.001), compared to women in the lowest quartile (8.0–20.5 kg; median, 17.6 kg). The magnitudes of associations with breast cancer risk per SD increases in BMI and body fat mass were similar with overlapping confidence intervals: RR 1.21; 95% CI 1.15–1.27 for BMI and RR 1.25; 95% CI 1.19–1.31 for body fat mass in multivariable‐adjusted models (Tables 3 and 4). Recoding quartiles using the corresponding DXA instead of baseline medians for tests of linear trend produced nearly identical results (Supporting Information Table S1).

**Table 3:** Association of height and overall adiposity measures with invasive breast cancer risk among never and former postmenopausal hormone replacement therapy users

					Stratified only	Multivariable‐adjusted	Further adjusted for body size at age 10	Further adjusted for body size at age 10
Height quartiles	Range (cm)	Median	Repeat median	Cases *n*	RR (95% CI)^a^	RR (95% CI)^b^	RR (95% CI)^d^	RR (95% CI)^d^
1	121–158	155	155	800	1	1	1	**Per SD increase**
2	158.1–162	161	160	720	1.07 (0.97‐1.18)	1.07 (0.96‐1.18)	1.07 (0.97‐1.18)	1.10 (1.05‐1.15)
3	162.1–166	164	164	653	1.09 (0.98‐1.20)	1.08 (0.97‐1.20)	1.08 (0.97‐1.20)	
4	166.1–193	169	169	740	1.30 (1.18‐1.44)	1.28 (1.16‐1.42)	1.27 (1.15‐1.41)	
χ^2^								175.7

**Weight quartiles**	**Range (kg)**	**Median**	**Repeat median**	**Cases *n***	**RR (95% CI)** a	**RR (95% CI)** c	**RR (95% CI)** d	**RR (95% CI)** d
1	34.0–61.9	57.4	57.1	565	1	1	1	**Per SD increase**
2	62.0–69.2	65.6	65.4	715	1.27 (1.14‐1.42)	1.25 (1.12‐1.40)	1.27 (1.13‐1.42)	1.24 (1.18‐1.30)
3	69.3–78.5	73.4	72.6	738	1.31 (1.18‐1.47)	1.29 (1.15‐1.44)	1.32 (1.18‐1.48)	
4	78.6–179.0	86.9	85.6	895	1.61 (1.45‐1.79)	1.58 (1.41‐1.76)	1.66 (1.48‐1.86)	
χ^2^								254.9

**BMI quartiles**	**Range (kg/m^2^)**	**Median**	**Repeat median**	**Cases *n***	**RR (95% CI)** a	**RR (95% CI)** c	**RR (95% CI)** d	**RR (95% CI)** d
1	14.6–23.7	22.2	22.2	579	1	1	1	**Per SD increase**
2	23.8–26.4	25.1	25.1	743	1.25 (1.12‐1.39)	1.26 (1.13‐1.41)	1.28 (1.15‐1.43)	1.21 (1.15‐1.27)
3	26.5–29.9	28	28	753	1.29 (1.15‐1.43)	1.32 (1.18‐1.47)	1.35 (1.21‐1.51)	
4	30.0–68.1	33.1	33	838	1.45 (1.30‐1.62)	1.52 (1.36‐1.70)	1.60 (1.43‐1.79)	
χ^2^								242.6

**Body fat mass quartiles**	**Range (kg)**	**Median**	**Repeat median**	**Cases *n***	**RR (95% CI)** a	**RR (95% CI)** c	**RR (95% CI)** d	**RR (95% CI)** d
1	8.0–20.5	17.3	17.6	537	1	1	1	**Per SD increase**
2	20.6–25.8	23.2	23.4	727	1.33 (1.19‐1.49)	1.31 (1.17‐1.47)	1.32 (1.18‐1.48)	1.25 (1.19‐1.31)
3	25.9–32.5	28.8	28.8	756	1.37 (1.23‐1.53)	1.35 (1.21‐1.51)	1.38 (1.23‐1.54)	
4	32.6–108.4	38.5	37.6	893	1.64 (1.48‐1.83)	1.62 (1.45‐1.82)	1.70 (1.52‐1.90)	
χ^2^								260.3

**Body fat % quartiles**	**Range (%)**	**Median**	**Repeat median**	**Cases *n***	**RR (95% CI)** a	**RR (95% CI)** c	**RR (95% CI)** d	**RR (95% CI)** d
1	9.4–32.9	29.7	30.5	550	1	1	1	**Per SD increase**
2	33.0–37.4	35.4	35.7	729	1.30 (1.16‐1.45)	1.29 (1.15‐1.44)	1.30 (1.16‐1.45)	1.23 (1.17‐1.29)
3	37.5–41.8	39.5	39.8	763	1.33 (1.19‐1.48)	1.32 (1.18‐1.48)	1.34 (1.20‐1.50)	
4	41.9–69.8	44.8	44.6	871	1.54 (1.38‐1.72)	1.55 (1.39‐1.73)	1.60 (1.43‐1.79)	
χ^2^								249.4

a Stratified by age at recruitment, region of recruitment and socioeconomic status (Townsend deprivation index).

b Adjusted for family history of breast cancer, age at menarche, age at first birth, parity, age at menopause, previous HRT use, smoking, alcohol intake frequency, physical activity and ethnicity.

c Adjusted for family history of breast cancer, age at menarche, age at first birth, parity, age at menopause, previous HRT use, smoking, alcohol intake frequency, physical activity ethnicity and height.

d Further adjusted for body size at age 10.

**Table 4:** Association of central adiposity measures and invasive breast cancer risk among never and former postmenopausal HRT users

					Stratified only	Multivariable‐adjusted	Further adjusted for body size at age 10	Further adjusted for body size at age 10	Further adjusted for body fat mass
Waist quartiles	Range (cm)	Median	Repeat median	Cases *n*	RR (95% CI)^a^	RR (95% CI)^b^	RR (95% CI)^c^	RR (95% CI)^c^	RR (95% CI)^d^
1	48–76	72	74	576	1	1	1	**Per SD increase**	**Per SD increase**
2	76.1–84	81	83	757	1.23 (1.10–1.37)	1.22 (1.09–1.36)	1.23 (1.10–1.37)	1.23 (1.17–1.29)	1.07 (0.99–1.16)
3	84.1–93	89	90	750	1.33 (1.19–1.48)	1.31 (1.18–1.47)	1.33 (1.19–1.49)		
4	93.1–171	101	100	830	1.52 (1.37–1.69)	1.51 (1.36–1.69)	1.57 (1.41–1.76)		
*χ* ^2^								246.3	

Hip quartiles	Range (cm)	Median	Repeat median	Cases *n*	RR (95% CI)^1^	RR (95% CI)^2^	RR (95% CI)^3^	RR (95% CI)^3^	RR (95% CI)^4^
1	62.0–97.0	94	95	658	1	1	1	**Per SD increase**	**Per SD increase**
2	97.1–102.0	100	101	693	1.31 (1.17–1.45)	1.28 (1.15–1.42)	1.29 (1.16–1.44)	1.21 (1.15–1.27)	1.01 (0.92–1.10)
3	102.1–109	105	106	772	1.35 (1.22–1.50)	1.32 (1.19–1.47)	1.34 (1.21–1.50)		
4	109.1–179	116	116	790	1.50 (1.35–1.66)	1.47 (1.32–1.63)	1.53 (1.38–1.71)		
*χ* ^2^								235.8	

WHR quartiles	Range	Median	Repeat median	Cases *n*	RR (95% CI)^1^	RR (95% CI)^2^	RR (95% CI)^3^	RR (95% CI)^3^	RR (95% CI)^4^
1	0.503–0.773	0.745	0.771	673	1	1	1	**Per SD increase**	**Per SD increase**
2	0.775–0.820	0.798	0.817	680	0.99 (0.89–1.11)	1.0 (0.90–1.11)	1.00 (0.90–1.11)	1.14 (1.08–1.21)	1.03 (0.97–1.10)
3	0.820–0.870	0.843	0.848	718	1.03 (0.93–1.15)	1.04 (0.94–1.16)	1.05 (0.94–1.17)		
4	0.870–1.351	0.905	0.889	842	1.23 (1.11–1.37)	1.25 (1.13–1.39)	1.27 (1.14–1.41)		
*χ* ^2^								202.0	

**Trunk fat mass quartiles**	**Range (kg)**	**Median**	**Repeat median**	**Cases *n***	**RR (95% CI)** d	**RR (95% CI)** a	**RR (95% CI)** b	**RR (95% CI)** b	
**1**	0.6–10.2	8.4	8.6	545	1	1	1	**Per SD increase**	
**2**	10.3–13.3	11.8	12	731	1.29 (1.15‐1.44)	1.27 (1.13‐1.42)	1.28 (1.14‐1.43)	1.25 (1.19‐1.31)	
**3**	13.4–16.9	15	14.8	741	1.33 (1.19‐1.48)	1.30 (1.16‐1.46)	1.33 (1.18‐1.49)		
**4**	17.0–48.3	19.9	19.3	896	1.66 (1.49‐1.84)	1.62 (1.45‐1.81)	1.69 (1.51‐1.89)		
**χ^2^**								259.4	

**Trunk fat % quartiles**	**Range (%)**	**Median**	**Repeat median**	**Cases *n***	**RR (95% CI)** d	**RR (95% CI)** a	**RR (95% CI)** b	**RR (95% CI)** b	
**1**	3.0–30.0	26.2	27.2	540	1	1	1	**Per SD increase**	
**2**	30.1–35.2	32.8	33.5	739	1.33 (1.19‐1.48)	1.31 (1.17‐1.47)	1.32 (1.18‐1.47)	1.25 (1.18‐1.31)	
**3**	35.3–40.0	37.5	37.6	753	1.36 (1.22‐1.52)	1.34 (1.20‐1.50)	1.36 (1.21‐1.52)		
**4**	40.1–75.6	43.3	42.5	881	1.61 (1.45‐1.80)	1.59 (1.42‐1.77)	1.63 (1.46‐1.83)		
**χ^2^**								250.9	

a Stratified by age at recruitment, region of recruitment and socioeconomic status (Townsend deprivation index).

b Adjusted for family history of breast cancer, age at menarche, age at first birth, parity, age at menopause, previous HRT use, smoking, alcohol intake frequency, physical activity, height and ethnicity.

Further adjusted for body size at age 10.

Further adjusted for body fat mass.

All indicators of central obesity were significantly associated with postmenopausal breast cancer risk (Fig. 1, Table 4); fat mass was slightly more informative about postmenopausal breast cancer risk (*χ* ^2^ = 259.4), and waist‐to‐hip ratio was least informative (*χ* ^2^ = 202.0). After adjusting for body fat mass, associations of breast cancer risk with per SD increases in waist circumference, hip circumference and waist‐to‐hip ratio were attenuated to the null. However, the confidence intervals around the adjusted associations were moderately wide so small residual associations cannot be ruled out. Results using standard categories for BMI and waist circumference^19^ are provided in Supporting Information Table S2. The results of sensitivity analyses excluding participants with any missing values did not differ materially from the main findings.

The magnitudes of risk associated with all measures of adiposity were greater in women who had been postmenopausal for 12 or more years; *p*‐values for interaction were <0.02 for all adiposity variables except waist‐to‐hip ratio, BMI and body and trunk fat percentage (Table 5). For instance, comparing the top to the bottom quartile of body fat mass, postmenopausal women who were at least 12 years since menopause had a greater increased risk of breast cancer (RR, 1.86; 95% CI, 1.56–2.21) compared to those who were <12 years since menopause (RR, 1.47; 95% CI 1.24–1.75).

**Table 5:** Association of body size/composition indices with invasive breast cancer risk in women who have been postmenopausal for <12 years *vs*. 12 or more years

	<12 years since menopause			≥12 years since menopause		
	Quartile	Cases, *n*	HR (95% CI)	Quartile	Cases, *n*	HR (95% CI)
Weight	1	288	1	1	283	1
	2	324	1.18 (0.99–1.40)	2	331	1.31 (1.10–1.55)
	3	284	1.05 (0.88–1.26)	3	387	1.56 (1.32–1.85)
	4	339	1.35 (1.14–1.61)	4	386	1.80 (1.52–2.13)
	*p* for interaction = 0.0049					
Body mass index	1	332	1	1	273	1
	2	317	1.10 (0.93–1.30)	2	357	1.40 (1.18–1.66)
	3	298	1.18 (1.00–1.41)	3	373	1.49 (1.26–1.77)
	4	298	1.28 (1.08–1.53)	4	383	1.83 (1.54–2.17)
	*p* for interaction = 0.0702					
Body fat mass	1	292	1	1	255	1
	2	326	1.25 (1.06–1.49)	2	336	1.39 (1.16–1.66)
	3	266	1.08 (0.90–1.29)	3	388	1.66 (1.40–1.97)
	4	337	1.47 (1.24–1.75)	4	383	1.86 (1.56–2.21)
	*p* for interaction = 0.0009					
Body fat percentage	1	309	1	1	255	1
	2	315	1.19 (1.01–1.41)	2	336	1.36 (1.14–1.62)
	3	290	1.18 (0.99–1.40)	3	373	1.50 (1.26–1.79)
	4	307	1.36 (1.15–1.62)	4	398	1.79 (1.51–2.13)
	*p* for interaction = 0.1106					
Waist	1	322	1	1	251	1
	2	340	1.16 (0.99–1.37)	2	373	1.37 (1.15–1.63)
	3	278	1.13 (0.95–1.35)	3	390	1.64 (1.38–1.94)
	4	296	1.32 (1.11–1.57)	4	374	1.83 (1.54–2.18)
	*p* for interaction = 0.0108					
Hip	1	336	1	1	312	1
	2	299	1.17 (0.99–1.38)	2	342	1.49 (1.26–1.76)
	3	305	1.13 (0.96–1.34)	3	385	1.62 (1.37–1.90)
	4	296	1.29 (1.09–1.53)	4	349	1.73 (1.46–2.04)
	*p* for interaction = 0.0167					
Waist‐to‐hip ratio	1	346	1	1	300	1
	2	314	1.02 (0.86–1.20)	2	325	1.04 (0.88–1.23)
	3	277	0.97 (0.81–1.14)	3	350	1.12 (0.95–1.33)
	4	299	1.17 (0.99–1.38)	4	413	1.36 (1.16–1.60)
	*p* for interaction = 0.4025					
Trunk fat mass	1	295	1	1	254	1
	2	319	1.20 (1.01–1.43)	2	348	1.37 (1.15–1.64)
	3	269	1.08 (0.90–1.29)	3	379	1.61 (1.35–1.91)
	4	336	1.43 (1.21–1.70)	4	381	1.87 (1.57–2.23)
	*p* for interaction = 0.0094					
Trunk fat percentage	1	294	1	1	255	1
	2	320	1.25 (1.05–1.48)	2	346	1.37 (1.14–1.63)
	3	293	1.22 (1.02–1.45)	3	361	1.51 (1.27–1.80)
	4	313	1.42 (1.19–1.69)	4	400	1.82 (1.53–2.16)
	*p* for interaction = 0.1580					

All analyses adjusted for family history of breast cancer, age at menarche, age at first birth, parity, age at menopause, previous HRT use, smoking, alcohol intake frequency, physical activity, height, ethnicity and body size at age 10.

## Discussion

In this large prospective study of postmenopausal women, we found statistically significant positive associations between all measures of adiposity and breast cancer risk. The relative risks, comparing the top with the bottom quartiles, were marginally greater for fat mass measured by impedance, but the RRs per SD increase and *χ* ^2^ statistics associated with each index of adiposity were of similar magnitude. All measures of central adiposity were also associated with postmenopausal breast cancer risk but these associations were attenuated after adjusting for body fat mass. Associations across all measures of adiposity were stronger in women who had been postmenopausal for ≥12 years.

Previous studies using impedance measures (based on 12,159; 7,523 and 13,598 participants) demonstrated a moderately greater magnitude of risk associated with postmenopausal breast cancer when comparing body fat to its surrogate measure, BMI.^20^, ^21^, ^22^ Another study using DXA measures with 503 incident breast cancer cases concluded that there is no difference in the ability to predict postmenopausal breast cancer risk when comparing anthropometric indices to DXA‐derived measures of body fat.^23^ Our results are consistent with the findings from the latter study in that BMI and waist circumference were as informative for breast cancer risk as impedance measures of adiposity.

In these data, all measures of adiposity showed stronger associations with postmenopausal breast cancer risk among women who were ≥12 years since menopause compared to women with <12 years since menopause. These findings are consistent with those from two previous large prospective studies which found that the positive association between adiposity and postmenopausal breast cancer risk was more marked in women aged 65 or older^6^ and in women who had experienced menopause ≥15 years previously.^22^

Elevated estrogen levels may stimulate carcinogenesis through increased cell proliferation as well as through pro‐angiogenic and anti‐apoptotic effects.^3^, ^24^ The relationship of adiposity with breast cancer risk in postmenopausal women can largely be explained by increased endogenous estrogen and decreased sex hormone‐binding globulin. In two large studies with prospectively measured estrogen, adjustment for free estradiol completely attenuated the association between adiposity and breast cancer risk, suggesting that this relationship may be mediated by the concentration of bioavailable estradiol.^25^, ^26^ Our results further suggest that the excess risk associated with increased levels of endogenous estrogens may take several years to fully outweigh the reduction in risk associated with adiposity in premenopausal women.^13^, ^27^

A 2003 review on central obesity found that waist circumference and waist‐to‐hip ratio were no longer associated with postmenopausal breast cancer risk after adjusting for BMI or weight,^28^ and some subsequent studies showed similar results.^13^, ^29^, ^30^ However, a recent meta‐analysis found that the association between waist circumference and postmenopausal breast cancer risk was only slightly attenuated after adjusting for BMI.^9^ In our analyses, which were adjusted for measured body fat mass rather than BMI, all associations between postmenopausal breast cancer and anthropometric measures of central adiposity were attenuated after adjustment for overall body fat, suggesting that overall body fat is the underlying adiposity‐related predictor of postmenopausal breast cancer risk.

Recent Mendelian randomization studies report an inverse association between BMI predicted using single nucleotide polymorphisms identified by genome‐wide association studies and postmenopausal breast cancer risk.^31^, ^32^ However, the BMI genetic score used in these studies may be more predictive of BMI in early‐life rather than later adulthood.^33^ In agreement with prior studies,^17^, ^34^ we found a protective association between greater childhood adiposity and breast cancer risk. We subsequently adjusted for comparative body size at age 10 as an independent risk factor for breast cancer and present relative risks both before and after this adjustment.

A notable strength of our study is the availability of objectively measured, rather than self‐reported, adult body size and composition measures. Self‐reported measures of adiposity such as weight tend to be underestimated, especially among overweight and obese women.^10^ Other strengths include our prospective design, large sample size and virtually complete follow‐up.^35^ The availability of a wide range of known and putative risk factors for breast cancer allowed us to address potential bias and confounding.

Bioelectrical impedance measurements have been validated against hydrodensitometry, which is regarded as the gold standard for measuring body fat.^36^, ^37^ Although bioelectrical impedance measurements capture body fat more accurately than anthropometric measurements,^8^, ^38^ they have limitations. Studies assessing bioelectrical impedance in diverse populations may require population‐specific calibration equations for different ethnic groups.^8^ This is of limited concern in our study since over 95% of participants are White and we have adjusted for ethnic group in our analyses. Hydration status can also affect impedance analysis.^39^ However, the accuracy of body composition measurements was improved by using a standardized measurement protocol administered by trained staff as well as utilizing repeat assessment data on all the body size and composition measures. Another limitation of the study is the lack of information on hormone receptor status of the tumor; however, since 70–80% of breast cancers included in our study of postmenopausal women are likely to be hormone receptor positive, the results are largely representative of hormonally responsive breast cancers.^40^

In conclusion, in this large prospective study, we found strong positive associations between all body size/composition variables and postmenopausal breast cancer risk. Anthropometric indices, with the exception of waist‐to‐hip ratio, were as informative for breast cancer risk as impedance measures of fat mass. Our results also suggest no association between postmenopausal breast cancer risk and abdominal adiposity beyond its contribution to overall adiposity.

## Supporting information

Supporting Information Figure 1

Supporting Information Table 1

Supporting Information Table 2
