# Supplementary figures and images for: Adiposity and breast cancer risk in postmenopausal women: Results from the UK Biobank prospective cohort
Source: Int J Cancer. 2018 Apr 10;143(5):1037–46. doi: 10.1002/ijc.31394 (PMC6099222; doi:10.1002/ijc.31394)

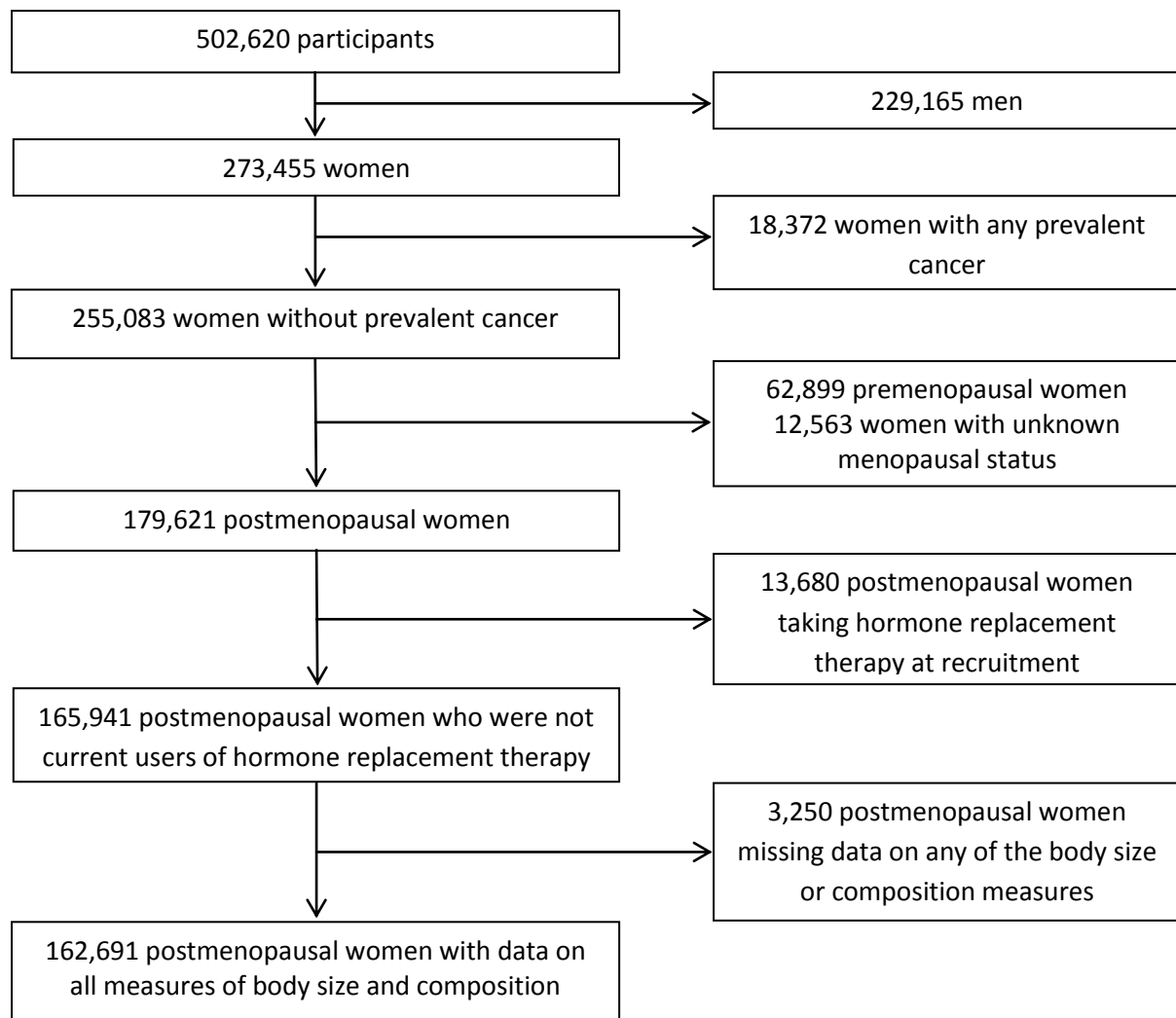

Supplement: Supplementary file 1 — Supporting Information Figure 1 [file IJC-143-1037-s001.pdf]
