# Supplementary material for: Adiposity and breast cancer risk in postmenopausal women: Results from the UK Biobank prospective cohort
Source: Int J Cancer. 2018 Apr 10;143(5):1037–46. doi: 10.1002/ijc.31394 (PMC6099222; doi:10.1002/ijc.31394)
Supplement: Supplementary file 2 — Supporting Information Table 1 [file IJC-143-1037-s002.doc]

**Supplementary Table 1**. Linear trend using baseline repeat measures compared to DXA measures

|  | **Using baseline measures** | **Using DXA measures** |
| --- | --- | --- |
|  |  |  |
| **Body fat mass, HR (95% CI) per 10 kg increase** | 1.25 (1.19-1.31) | 1.27 (1.20-1.33) |
|  |  |  |
| **Body fat percentage, HR (95% CI) per 10% increase** | 1.34 (1.25-1.44) | 1.36 (1.26-1.46) |
|  |  |  |
| **Trunk fat mass, HR (95% CI) per 10 kg increase** | 1.52 (1.39-1.67) | 1.52 (1.39-1.67) |
|  |  |  |
| **Trunk fat percentage, HR (95% CI) per 10% increase** | 1.31 (1.23-1.39) | 1.30 (1.22-1.38) |
|  |  |  |

| All analyses stratified by age at recruitment, region of recruitment, and Townsend deprivation index |
| --- |
| All analyses adjusted for adjusted for family history of breast cancer, age at menarche, age at first birth, parity, age at menopause, previous HRT use, smoking, alcohol intake frequency, physical activity, height, ethnicity, and body size at age 10 |
