# Supplementary material for: Adiposity and breast cancer risk in postmenopausal women: Results from the UK Biobank prospective cohort
Source: Int J Cancer. 2018 Apr 10;143(5):1037–46. doi: 10.1002/ijc.31394 (PMC6099222; doi:10.1002/ijc.31394)
Supplement: Supplementary file 3 — Supporting Information Table 2 [file IJC-143-1037-s003.doc]

**Supplementary Table 2. Association of BMI and waist circumference with invasive breast cancer risk, using standard categories**

|  |  |  |  | **Stratified only** | **Multivariable-adjusted** | **Further adjusted for body size at age 10** | **Further adjusted for body fat mass** |
| --- | --- | --- | --- | --- | --- | --- | --- |
| **BMI (kg/m^2^)** | **Median** | **Repeat median** | **Cases *n*** | **RR (95% CI)^a^** | **RR (95% CI)^b^** | **RR (95% CI)^c^** |  |
| <25.0 | 23.0 | 23.0 | 921 | 1 | 1 | 1 |  |
| 25.0-29.9 | 27.1 | 27.0 | 1,154 | 1.22 (1.12-1.33) | 1.24 (1.14-1.36) | 1.27 (1.16-1.38) |  |
| 30.0-34.9 | 31.9 | 31.8 | 548 | 1.32 (1.19-1.47) | 1.37 (1.23-1.53) | 1.43 (1.28-1.59) |  |
| ≥35.0 | 37.8 | 37.3 | 290 | 1.48 (1.29-1.69) | 1.56 (1.36-1.79) | 1.67 (1.45-1.92) |  |
|  |  |  |  |  |  |  |  |
| **Waist circumference (cm)** | **Median** | **Repeat median** | **Cases *n*** | **RR (95% CI)^a^** | **RR (95% CI)^b^** | **RR (95% CI)^c^** | **RR (95% CI)^d^** |
| <80.0 | 74.0 | 76.0 | 851 | 1 | 1 | 1 | 1 |
| 80.0-87.9 | 83.0 | 85.0 | 767 | 1.20 (1.09-1.32) | 1.19 (1.08-1.32) | 1.20 (1.09-1.33) | 1.10 (0.99-1.22) |
| ≥88.0 | 96.0 | 96.0 | 1,295 | 1.36 (1.24-1.48) | 1.35 (1.23-1.48) | 1.39 (1.27-1.52) | 1.11 (0.98-1.26) |
|  |  |  |  |  |  |  |  |

| ^a^ stratified by age at recruitment, region of recruitment, and socioeconomic status (Townsend deprivation index) |
| --- |
| ^b^ adjusted for family history of breast cancer, age at menarche, age at first birth, parity, age at menopause, previous HRT use, smoking, alcohol intake frequency, physical activity, height, and ethnicity |
| ^c^ further adjusted for body size at age 10 |
| ^d^ further adjusted for body fat mass |
